# Supplementary material for: Maternal Experience of Domestic Violence, Associations with Children’s Lipid Biomarkers at 10 Years: Findings from MINIMat Study in Rural Bangladesh
Source: Nutrients. 2019 Apr 23;11(4):910. doi: 10.3390/nu11040910 (PMC6520974; doi:10.3390/nu11040910)
Supplement: Supplementary file 1 [file nutrients-11-00910-s001.pdf]

Table S1. Association of children's level of lipid biomarkers with maternal experience of any emotional DV before and after childbirth.

Biomarkers	Model	Maternal experience of any emotional DV			
		No experience	Before childbirth	After childbirth	Both before and after child birth
			ß (95% CI)	ß (95% CI)	ß (95% CI)
Apo A (g/l)	Unadjusted	Ref	-0.03 (-0.07, 0.01)	-0.00 (-0.03, 0.02)	-0.02 (-0.05, 0.01)
	Adjusted[1]	Ref	-0.03 (-0.06, 0.01)	-0.00 (-0.03, 0.03)	-0.01 (-0.05, 0.02)
Apo B (g/l)	Unadjusted	Ref	-0.02 (-0.05, 0.02)	0.00 (-0.02, 0.02)	-0.02 (-0.05, 0.01)
	Adjusted[1]	Ref	-0.02 (-0.05, 0.02)	-0.00 (-0.02, 0.02)	-0.03 (-0.05, 0.00)
Apo B/Apo A	Unadjusted	Ref	-0.01 (-0.04, 0.03)	0.00 (-0.02, 0.02)	-0.01 (-0.04, 0.01)
	Adjusted[1]	Ref	-0.02 (-0.04, 0.01)	-0.00 (-0.03, 0.02)	-0.01 (-0.04, 0.02)
HDL (mmol/l)	Unadjusted	Ref	-0.03 (-0.08, 0.01)	-0.01 (-0.04, 0.02)	-0.02 (-0.06, 0.02)
	Adjusted[1]	Ref	-0.03 (-0.08, 0.02)	-0.01 (-0.04, 0.02)	-0.02 (-0.06, 0.02)
LDL (mmol/l)	Unadjusted	Ref	-0.03 (-0.17, 0.10)	-0.03 (-0.12, 0.07)	0.03 (-0.08, 0.15)
	Adjusted[1]	Ref	-0.03 (-0.17, 0.10)	-0.02 (-0.12, 0.08)	0.04 (-0.08, 0.16)
LDL/HDL	Unadjusted	Ref	0.03 (-0.12, 0.18)	-0.00 (-0.11, 0.10)	0.08 (-0.05, 0.20)
	Adjusted[1]	Ref	0.02 (-0.13, 0.17)	0.00 (-0.10, 0.10)	0.07 (-0.06, 0.20)
Cholesterol (mmol/l)	Unadjusted	Ref	-0.09 (-0.23, 0.04)	-0.03 (-0.12, 0.07)	-0.05 (-0.17, 0.07)
	Adjusted[1]	Ref	-0.09 (-0.23, 0.05)	-0.02 (-0.12, 0.08)	-0.03 (-0.16, 0.09)
Triglycerides[2] (mmol/l)	Unadjusted	Ref	-0.01 (-0.08, 0.06)	0.02 (-0.04, 0.06)	-0.03 (-0.09, 0.03)
	Adjusted[1]	Ref	-0.02 (-0.09, 0.05)	0.01 (-0.04, 0.06)	-0.04 (-0.10, 0.02)

[1] Models adjusted for maternal education, SES and age. ß and 95% of CI obtained using general linear models

[2] The level of biomarker was transformed using the natural logarithm, and effect estimate reported accordingly. DV, Domestic violence; HDL, High Density Lipoprotein; LDL, Low Density Lipoprotein; Ref, reference category

Formatted: English (United States)
